# Differential expression of telomerase reverse transcriptase (hTERT) in lung tumours

**DOI:** 10.1038/sj.bjc.6601643

**Published:** 2004-02-24

**Authors:** S Lantuejoul, J C Soria, D Moro-Sibilot, L Morat, S Veyrenc, P Lorimier, P Y Brichon, L Sabatier, C Brambilla, E Brambilla

**Affiliations:** 1 Service de Pathologie Cellulaire, Institut A Bonniot, CHU Michallon Grenoble, France; 2 Lung Cancer Research Group INSERM U 578, Institut A Bonniot, CHU Michallon Grenoble, France; 3 Laboratoire de Radiobiologie et Oncologie DSV-DRR CEA Fontenay aux Roses, France

**Keywords:** lung cancer, hTERT, telomerase, immunohistochemistry, nucleolar localization

## Abstract

Human telomerase reverse transcriptase is a ribonucleoprotein that synthesises telomeric sequences, which decrease at each cell division. In cancer cells, its activity is linked to telomere maintenance leading to unlimited cellular proliferation and immortality. To evaluate the prognostic value of the catalytic subunit telomerase reverse transcriptase (hTERT), we analysed its expression by immunohistochemistry in 122 formalin-fixed lung tumours including 42 squamous cell carcinoma (SCC), 43 adenocarcinoma (ADC), 19 basaloid carcinoma (BC) and 18 small-cell lung carcinoma (SCLC) in comparison with detection of hTERT mRNA by *in situ* hybridisation and relative telomerase activity by TRAP assay in a subset of tumours. We observed a high concordance between hTERT protein expression and detection of hTERT mRNA and telomerase activity. Telomerase expression varied according to histology (*P*=0.0002) being significantly lower in ADC than in SCC, BC and SCLC (*P*<0.0001). Adenocarcinoma and SCC exhibited either a nuclear or a nucleolar staining in contrast with a diffuse nuclear staining observed in most BC and all SCLC (*P*=0.01). In stage I NSCLC telomerase expression was lower than in other stages (*P*=0.04), and a nucleolar staining was correlated with a short survival (*P*=0.03). We concluded that telomerase expression and pattern are distinctive among histopathological classes of lung cancer and convey prognostic influence.

Lung cancer represents the leading cause of cancer-related death in industrial countries and comprises about 20% small-cell lung carcinoma (SCLC) and 80% of non-small-cell lung carcinoma (NSCLC). According to the new WHO histological classification (Travis *et al*, 1999), non-small-cell lung carcinoma include with the common types squamous cell carcinoma (SCC) and adenocarcinoma (ADC), a recently described entity, the basaloid carcinoma (BC), as a variant of large cell carcinoma undergoing a particularly poor outcome (Brambilla *et al*, 1992). Surgical resection at early-stage disease represents the treatment of choice for NSCLC. However, survival rates remain low fostering identification of new prognostic factors and therapeutic target such as telomerase with the aim of deciphering new modes of adjuvant therapies.

Telomeres, which represent the end of the eukaryotic chromosomes, shorten at each cell division because of incomplete replication by DNA polymerase (Henderson, 1995). This results in telomere shortening leading to chromosome degradation or end fusion and cellular senescence acting as a ‘mitotic clock’ (Harley *et al*, 1990; Hastie *et al*, 1990; Allsopp *et al*, 1992). In germ line cells as well as in tumour cells, telomerase, a ribonucleoprotein complex composed of a reverse transcriptase catalytic subunit (hTERT) that copies a template region of RNA subunit (hTERC), can synthesise telomeric DNA, therefore, allowing cells to proliferate indefinitely (Counter *et al*, 1992; Nakamura and Cech, 1998; Holt and Shay 1999). While both hTERC and hTERT are required for telomerase activity, hTERC is expressed rather ubiquitously, whereas hTERT is the only limiting factor since its expression is confined to cells expressing telomerase activity (Meyerson *et al*, 1997; Nakamura *et al*, 1997; Kolquist *et al*, 1998).

Telomerase activity (TA) evaluated by a sensitive PCR-based telomere repeat amplification protocol (TRAP) assay has been widely reported in various malignancies such as liver, colorectal, brain, prostate and breast cancers as well as leukaemia (Bacchetti and Counter, 1995; Counter *et al*, 1995; Langford *et al*, 1995; Carey *et al*, 1998; Tahara *et al*, 1999; Kawakami *et al*, 2000). Regarding malignancies arising in the thorax, several studies have demonstrated a telomerase activity in SCLC and NSCLC carcinomas including NE tumours and adenocarcinomas and their precursor lesion (namely atypical alveolar hyperplasia) as well as in pulmonary sarcomas and mesotheliomas (Hiyama K *et al*, 1995; Ahrendt *et al*, 1997; Yashima *et al*, 1997; Gomez-Roman *et al*, 2000; Kumaki *et al*, 2001, 2002; Nakanishi *et al*, 2002). Almost all SCLC and the majority of NSCLC display a substantial telomerase activity in 62–96% of the cases (Hiyama K *et al*, 1995; Albanell *et al*, 1997; Gomez-Roman *et al*, 2000). Since a close relationship has been demonstrated between elevated telomerase activity and a poor prognosis in neuroblastoma and gastric carcinoma (Hiyama E *et al*, 1995a, 1995b), several reports have also suggested that high TA or high hTERT mRNA levels should be correlated with a poor survival in stage I NSCLC (Marchetti *et al*, 1999, 2002; Wang *et al*, 2002). High levels of telomerase have also been associated with tumour recurrence, histological type, grade (Marchetti *et al*, 1999, 2002; Kumaki *et al*, 2001) or smoking status (Xinarianos *et al*, 1999).

Since several reports have emphasised the use of noncommercial hTERT antibodies for telomerase analysis in lung cancer, mesothelioma, colon cancer and liver tissues (Tahara *et al*, 1999; Kawakami *et al*, 2000; Kumaki *et al*, 2001, 2002), we decided to test the specificity and the usefulness on a daily practice of a newly commercially available monoclonal hTERT antibody. In order to validate this immunohistochemical approach, we compared the analysis of hTERT protein expression in 122 lung tumours, with the results of a previously established hTERT *in situ* hybridisation technique (Soria *et al*, 2001) as well as with the standard TRAP assay performed in a subset of the same tumours. We also performed a comparative study of other commercially available hTERT antibodies using immunohistochemistry and Western blot. This enabled us to assess the value of hTERT immunohistochemistry and to show that aggressive lung tumours such as SCLC and basaloid carcinoma display the highest levels of hTERT expression, whereas the lowest levels are observed in adenocarcinoma and in stage I NSCLC.

## MATERIAL AND METHODS

### Patients and tissue samples

A total of 122 tumour specimens were collected from lung cancer resections or mediastinal biopsies performed for diagnosis. All samples were obtained within 1 h after surgical removal, and frozen tumour samples were stored at −80°C until protein extraction for TRAP assay and Western blotting along with standard formalin or Bouin fixation. Frozen samples were all analysed histologically and selected for protein extraction with the requirement of more than 70% of viable tumour cells and less than 10% of stromal lymphocytes. Histopathological and clinical data including pTNM stages according to the international UICC classification are listed in Table 1

**Table 1:** Summary of clinicopathologic features

**Variable**	**Number of cases**
Mean age; year	61 (range 39–84)
*Gender*	
Male	110
Female	12
	
*Stage (n*=*122)*	
I	34
II	29
III	55
IV	4
	
*Histological type (n*=*122)*	
SCC	42
ADC	43
BC	19
SCLC	18
	
*Metastases*	
Visceral	20/84 (24%)
Lymph node	61/122(50%)

.

Two normal lymph nodes, two normal and two inflammatory lung tissues with pneumonia were used as positive or negative controls for immunohistochemistry, *in situ* hybridisation, Western blotting and TRAP. Three cancer cell lines, obtained from the American Type Culture Collection (Manassas, VA, USA), NCI-H322 (lung ADC), NCI-H69 (SCLC) and HELA (cervical ADC) were also used as positive controls for Western blotting. The growing conditions were as follows: RPMI-1640 for NCI-H322, RPMI-1640 with 100 mM 1% sodium pyruvate for NCI-H69 and DMEM 10% for HELA, all media contained 10% foetal calf serum under 5% CO_2_.

### Immunohistochemical analysis

In total 3-*μ*m-thick sections were deparaffinised and pretreated for antigenic heat retrieving 1 h at 98°C with 10 mM citrate buffer pH 6. In order to compare different hTERT antibodies, a set of 10 normal and tumour samples were incubated 1–12 h at room temperature with primary polyclonal antibodies raised against hTERT protein (dilution 10 *μ*g ml^−1^): TRT-L20 (Santa Cruz Biotechnology, Santa Cruz, CA, USA), TEL-1 (Abcam, Cambridge, UK), TERT, Ab-2 and p123 (Calbiochem, San Diego, CA, USA). All the 122 tumour samples were also incubated 12 h at 42°C with a primary monoclonal hTERT 44F12 antibody (Novocastra, Newcastle upon Tyne, UK) at the dilution 1 : 20 (0.35 *μ*g *μ*l^−1^) on paraffin sections. Immunohistochemical analysis was also performed on frozen sections with this last antibody at the dilution 1 : 10 (0.7 *μ*g *μ*l^−1^) as control of negative cases. For both techniques, a three-stage indirect immunoperoxidase technique was performed on Nexes Ventana automated staining module (Tucson, AZ, USA), which enables a standardisation of reaction time and temperature, washing procedures and development of staining and amplification. Negative control consisted in omission of the primary antibody and incubation with immunoglobulins of the same species. The levels of protein expression were evaluated by two pathologists (SL and EB). The distribution of staining was graded as 0: absent, 1: <10%, 2: 10–50%, 3: 51–90% and 4: >90% and intensity was graded as 1: weak, 2: medium, and 3: strong. The combined score was performed as follow: score=distribution+intensity. Nuclear and/or nucleolar stainings were regarded as specific patterns.

### Western blot analysis

In all, 30 tumour samples including 13 SCC, 10 ADC, five BC and two SCLC, two normal lung specimen and NCI-H322, NCI-H69 and HELA cancer cell lines were solubilised in Laemmli buffer. Total proteins were quantified using the Bio-Rad kit (Bio-Rad, Hercules, CA, USA) and equal amounts of protein were located on a 7.5% SDS–PAGE gel. Gel was then transferred to nitrocellulose, which was preincubated with primary telomerase polyclonal antibodies TRT-L20 (Santa Cruz Biotechnology, Santa Cruz, CA, USA), TEL-1 (Abcam, Cambridge, UK), TERT, Ab-2 or p123 (Calbiochem, San Diego, CA, USA) or with telomerase monoclonal antibody 44F12, at the dilution 1 : 500 at 4°C overnight. On the next day, a anti-mouse biotinylated IgG was used for 1 h at room temperature and immunoreactive bands were visualised using the ECL^Plus^ detection system (Amersham, Amersham, UK) as suggested by the manufacturer. Stained bands when present were qualified as weak or strong.

### *In situ* hybridisation

For riboprobe generation and RNA *in situ* hybridisation, a TOPO TA cloning vector (PCR®II-TOPO; Invitrogen, Carlsbad, CA, USA) was used containing a 430 bp *Eco*RV–*Bam*H1 fragment of the hTERT cDNA as previously described (Soria *et al*, 2001) to generate a digoxigenin-labelled RNA probe (riboprobe) specific for the antisense strand of the hTERT cDNA, which hybridises to the full-length transcript corresponding to the catalytic domain of the enzyme.

*In situ* hybridisation was performed on paraffin-embedded tissue sections from 20 tumour samples including five ADC, seven BC, seven SCC and one SCLC, in RNAse-free conditions. The slides were transferred on the heating blocks of a Discovery module (Ventana Medical System, Strasbourg, France) for an automatised *in situ* hybridisation procedure. Briefly, the sections were treated with 2.5 *μ*g ml^−1^ proteinase K (Roche Diagnostics, Meylan, France) for 14 min at 37°C and postfixed in 4% paraformaldehyde for 8 min at room temperature. The antisense riboprobe diluted at 800 ng ml^−1^ in hybridisation buffer was denatured and 100 *μ*l applied on each sections for an incubation of 8 h at 42°C. Sense and antisense actin riboprobes have been used as negative and positive controls, respectively, to assess the good mRNA preservation in the same conditions on duplicate slides. Slides were then incubated overnight at room temperature with an alkaline phosphatase-conjugated antidigoxigenin antibody (dilution 1 : 200, Roche Diagnostics, Meylan, France). Alkaline phosphatase was detected using 5-bromo-4-chloro-3-indolyl phosphate and nitro-blue tetrazolium chloride (Roche Diagnostics, Meylan, France) as chromogens. The levels of hTERT mRNA expression were evaluated by two pathologists (SL and EB). The same score system than for immunohistochemistry data combining distribution and intensity of staining was applied. Cytoplasmic pattern was considered as a specific positive staining.

### TRAP assay

The kit Telo TAGGG telomerase PCR ELISA^plus^ (Roche Molecular Biochemicals, Mannheim, Germany), a photometric enzyme immunoassay, was used for quantitative determination of telomerase activity. Frozen sections from 40 tumour samples, two normal lymph nodes, two normal lungs and one with pneumonia were analysed histologically to assess the amount of tumour component (at least 70% of tumour cells), and the quality of material (i.e. absence of necrosis). All steps of the assay protocol were performed in RNAse-free conditions. In total, 20 10-*μ*m-thick frozen sections of each specimen were cut, immediately lysed in 200 *μ*l of ice-cold CHAPS (3-{[3-chlomidopropyl]-dimethyl-ammonio}-1-propanesulphonate) lysis buffer, homogenised, incubated on ice for 30 min and centrifuged at 12 000 **g** for 20 min at 4°C. In all, 2 *μ*l of each sample, with heat-inactivated controls, were mixed with 48 *μ*l of reaction mixture containing telomerase substrate, biotin labelled P1-TS and P2 primers, nucleotides and Taq polymerase in Tris buffer. Reverse transcription was performed for 30 min at 25°C and PCR amplification consisted in a three-step PCR at 94°C 30 s, 50°C 30 s and 72°C 90 s for 30 cycles followed by a 72°C 10-min extension step. Evaluation of the relative telomerase activity (RTA) was determined by the measurement of the absorbance of each sample at a wavelength of 450 nm (reference wavelength, 595 nm, Microplate Reader 3550, Bio Rad). Cases were considered as positive when RTA was higher than 0.2.

### Statistical analysis

The staining scores were compared in different categories using the Mann–Whitney *U* test and Kruskal–Wallis H tests. The *χ*^2^ test was used to test the association between two categorical variables. Survival times were calculated from the date of surgery from cancer-related events. Survival curves were estimated by the method of Kaplan–Meier. The log-rank test was used to compared survival curves. P-values less than 0.05 were considered as statistically significant. All the tests were performed with the Stat View program (Abacus Concepts, Berkeley, CA, USA)

## RESULTS

### Immunohistochemical analysis of human TERT

Using TRT-L20 and TEL-1 polyclonal antibodies, a nuclear staining was observed in tumour cells and activated lymphocytes but also in stromal and epithelial normal cells. With TERT and Ab-2 polyclonal antibodies, nuclear staining was observed in tumour cells whereas activated lymphocytes and normal cells remained negative. All cells exhibited a strong cytoplasmic staining. In contrast, no nuclear staining could be demonstrated with p123 polyclonal antibody regardless of cell type.

**Figure 1:**
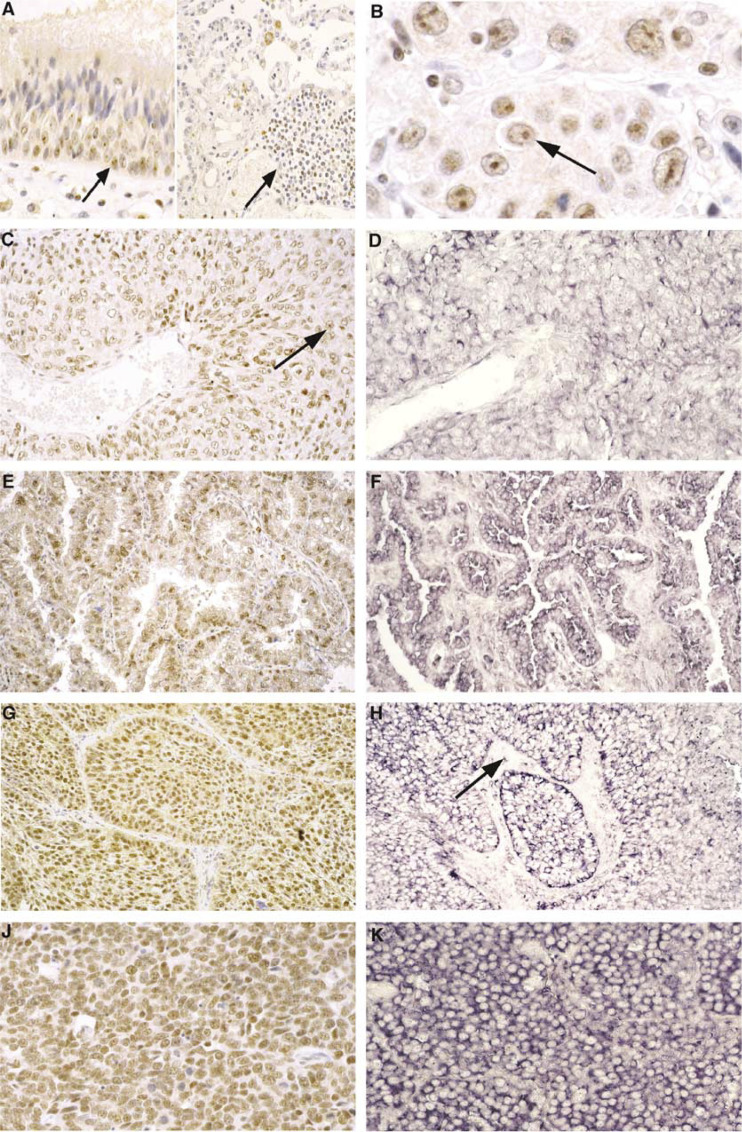
Expression in nontumoral lung and in lung tumours of hTERT as compared with hTERT mRNA by immunohistochemistry and *in situ* hybridisation. (**A**) (left): Mild to moderate hTERT immunostaining (intensity 1) of basal cells of normal bronchial epithelium with nucleolar reinforcement (arrow) and moderate staining (intensity 2) of activated lymphocytes in bronchial mucosae (immunoperoxidase staining, × 200). (**A**) (right): hTERT positive staining in a lymphocytic aggregate (arrow) in an inflammatory lung. Alveolar epithelial cells are negative (immunoperoxidase staining, × 100). (**B**) A typical nucleolar staining (arrow) in an ADC (immunoperoxidase staining, × 400). (**C**) hTERT immunostaining in SCC exhibiting a strong nucleolar staining (intensity 3) (arrow) (immunoperoxidase staining, × 100). (**D**) mRNA expression observed by *in situ* hybridisation in the cytoplasm of tumoral cells and stromal lymphocytes in the same case as 1C (× 100). (**E**) hTERT immunostaining in ADC exhibiting a strong nuclear staining (intensity 3) (immunoperoxidase staining, × 100). (**F**) mRNA expression in the same case as 1E shown by *in situ* hybridisation (× 100). (**G**) hTERT immunostaining in BC showing a strong and diffuse nuclear staining (intensity 3) (immunoperoxidase staining, × 100). (**H**) mRNA expression shown by *in situ* hybridisation in the cytoplasm of tumour cells and stromal lymphocytes (arrow) in the same case of BC (× 100). (**J**) Strong and diffuse nuclear hTERT immunostaining (intensity 3) in a SCLC (immunoperoxidase staining, × 200). (**K**) mRNA expression shown by *in situ* hybridisation in the same case as **J** (× 200).

Using monoclonal antibody 44F12, a mild to moderate nuclear immunostaining with nucleolar reinforcement (intensity 1 or 2) was observed on about 50% of basal cells of normal bronchial epithelium (Figure 1A

, left). Serous cells of bronchial glands also displayed a frequent focal nucleoplasmic and nucleolar-positive staining, whereas alveolar pneumonocytes and endothelial cells remained negative (Figure 1A, right). In contrast, 50–80% of lymphocytes present in the bronchial mucosae or infiltrating alveolar walls in inflammatory lungs (Figure 1A) showed a moderate nuclear staining and served as internal positive control with a staining intensity of 2 for grading the intensity of staining in neoplastic cells (from 1 to 3).

Among 122 cases of lung tumours, 116 were hTERT positive (95%) with 44F12 monoclonal antibody. This included all cases of SCLC, BC and SCC and 86% of adenocarcarcinomas (37 out of 43) (Figure 1C, E, G, J). All results concerning scores and staining pattern of hTERT expression in lung tumours are summarised in Table 2

**Table 2:** Differential immunohistochemical hTERT expression in lung tumours (scores and pattern of staining)

**Histology (nb of cases) (*n*=122)**	**Distribution of scores: nb of cases (%)**				**Staining pattern distribution: nb of cases**	
	**0**	**2 and 3**	**4 and 5**	**6 and 7**	**Nuclear**	**Nucleolar**
ADC (43)	6 (14%)	17 (**40**%)	18 (**42**%)	2 (4%)	**16**	**12**
SCC (42)		7 (16.6%)	28 (**66.6**%)	7 (16.6%)	**17**	**14**
BC (19)		2 (10%)	13 (**68**%)	4 (**21**%)	**14**	**5**
SCLC (18)		1 (5%)	13 (**72**%)	4 (**22**%)	**18**	

. Positive staining in tumour cells was located on nucleus, either restricted to nucleolar structures (Figure 1B), or diffuse in the nucleoplasm with nucleolar reinforcement. The first pattern, exclusively nucleolar, was more frequently observed in SCC (14 out of 42; 33%) and in ADC (16 out of 43; 37%) than in BC (5 out of 19; 26%), and SCLC (none) (*P*=0.01) (*χ*^2^ test). We did not observe a nucleolar exclusion in cases with diffuse nuclear pattern.

Tumoral heterogeneity in hTERT expression distribution was frequent, whatever the histological type of tumour considered. Furthermore, ADC exhibited either low or moderate levels of hTERT expression in contrast with BC, SCLC and SCC presenting moderate to high levels of telomerase expression. Moreover, in six mixed type ADC with predominant BAC peripheral pattern (lepidic growth), only three exhibited telomerase expression in the lepidic growth. The distribution of the levels of telomerase expression among histological type were statistically different (*P*=0.0002) (Kuskal–Wallis H test), and hTERT scores were significantly lower in ADC than in SCC, BC and SCLC taken together (*P*<0.0001) (*χ*^2^ test).

### hTERT Western blotting

With polyclonal antibody p123, numerous nonspecific bands were seen on lysates from cell lines, normal or tumour samples. In contrast, with TRT-L20, TEL-1, TERT and Ab-2 antibodies, no specific stained band was observed using several cell line lysates or from tumour samples.

**Figure 2:**
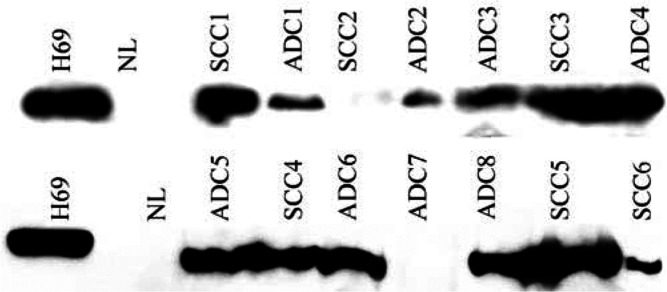
Western Blotting showing a 105 kDa hTERT product with hTERT 44F12 antibody in cancer cell line H69, normal lung (NL) and a subset of lung carcinoma including SCC and ADC

Using 44F12 antibody, all cancer cell lines strongly exhibited a positive and unique band of approximately 105 kDa consistent with the theoretical estimation of telomerase molecular weight of 127 kDa (Wick *et al*, 1999). This band was totally absent in two normal lung samples. Among lung cancers, 24 out of 30 cases (80%) displayed an hTERT expression with a unique band of 105 kDa, 17 cases exhibiting a strong staining and seven a weak expression (Figure 2

). One SCLC and one BC exhibited a double band both located around 105 kDa (data not showed), but this phenomenon was too rare to enable us any conclusion about a possible phosphorylation.

### *In situ* Hybridisation with hTERT riboprobe

*In situ* hybridisation staining with hTERT riboprobe was located in the cytoplasm and had the same cell distribution than that obtained by immunohistochemistry. It was negative in most normal lung structures but positive in a few basal bronchial cells, serous cells of bronchial glands and lymphocytes. Hybridisation with sense actin riboprobe gave an expected negative signal in all cell type.

All 20 lung tumours were strongly positive, with no staining on stromal cells except activated lymphocytes (Figure 1 D, F, H, K). The scores of expression ranged from 4 to 6; nevertheless, the small number of cases precluded demonstration of a statistical significance according to histological types.

### TRAP assay

In two normal lungs, two with pneumonia and two normal lymph nodes, RTA remained negative. In all, 34 out of 40 cases of lung tumours (85%) of all histological types exhibited levels of RTA higher than 0.2, including 64% of ADC, 64% of SCC, 92% of the BC, and the SCLC case. The RTA levels were significantly higher in BC (1.28±0.69 RTA) than in SCC (0.68±0.84 RTA) and in ADC (1.09±0.58) (*P*=0.03 BC *vs* SCC and ADC considered together). The only SCLC case studied exhibited a very high RTA (1.789 RTA).

### Concordance between data obtained by Western blotting and by immunohistochemistry (Table 3)

**Table 3:** Comparison of Western blotting staining intensity and immunohistochemical scores of hTERT expression in a subset of 30 lung tumours including 13 SCC, 10 ADC, five BC and two SCLC

**Histology**	**Western blotting**	**Immunohistohemical scores**
SCC 1	Strong	6
SCC 2	Negative	3
SCC 3	Strong	5
SCC 4	Strong	4
SCC 5	Strong	6
SCC 6	Weak	4
SCC 7	Negative	4
SCC 8	Weak	3
SCC 9	Strong	4
SCC 10	Weak	4
SCC 11	Strong	5
SCC 12	Strong	5
SCC 13	Strong	5
ADC 1	Weak	3
ADC 2	Weak	4
ADC 3	Weak	3
ADC 4	Strong	4
ADC 5	Strong	5
ADC 6	Strong	0
ADC 7	Negative	3
ADC 8	Strong	5
ADC 9	Weak	3
ADC 10	Strong	5
BC 1	Strong	7
BC 2	Strong	5
BC 3	Negative	3
BC 4	Negative	3
BC 5	Weak	6
SCLC 1	Weak	4
SCLC 2	Strong	5

Weak or negative stained bands correlated with immunohistochemical scores lower than score 4 in eight cases. Conversely, a strong Western blot staining was observed in 16 cases presenting immunohistochemical scores of 4–7. One case presented a strong WB staining despite a negative immunohistochemical staining (ADC6), and six cases exhibited a negative or weak WB staining despite high scores of HTERT expression by immunohistochemistry (SCC6, SCC7, SCC10, ADC2, BC5 and SCLC1). Overall, a concordance between data obtained by immunohistochemistry and Western blotting was of 76%.

### Concordance between data obtained by immunohistochemistry, *in situ* hybridisation, and TRAP

Among 40 cases analysed concomitantly by TRAP and immunohistochemistry, 34 (85%) were positive with both techniques. In the six TRAP negative tumours, no RTA was detected despite hTERT protein expression in five cases and one case had neither RTA nor protein expression. In all, 24 tumours exhibited either low levels of RTA (⩽0.5) and low hTERT scores 2 or 3, or high levels of RTA (more than 0.5) and high hTERT combined scores of 4–7 in concordance. Conversely, 10 cases were discordant: four cases showed low RTA but high hTERT staining scores of 5 or 6, and six cases high RTA but low hTERT staining score 3. Overall, a concordance of 70% between the levels of activity and scores of hTERT immunohistochemical expression was observed.

Among these 40 cases, 18 were concomitantly studied by *in situ* hybridisation and immunohistochemistry, showing that protein expression was always observed when hTERT mRNA and telomerase activity were measurable. Among 20 cases studied by both *in situ* hybridisation and immunohistochemistry, six cases exhibited high levels of hTERT mRNA (scores 5 or 6) and a low protein staining score of 3. Concordance between immunohistochemical analysis and *in situ* hybridisation was of 70%. Nucleolar staining was also observed when telomerase activity was detected (in 10 tumours), or mRNA were present (five cases) or both (five cases), as well as when a specific band was observed in Western blot (five cases).

### Correlation between telomerase expression and clinicopathologic features

**Figure 3:**
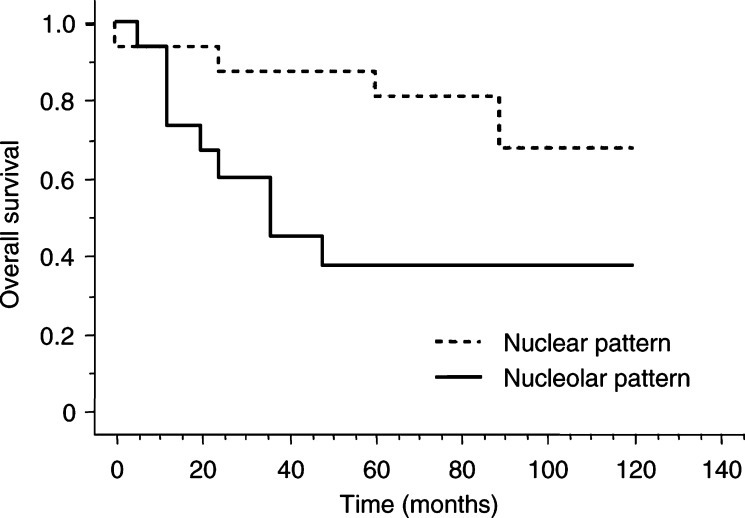
Survival curve of stage I NSCLC patients according to subcellular location of hTERT immunohistochemical staining (*P*=0.03).

Immunohistochemical or *in situ* hybridisation staining scores or RTA values were not correlated with gender or patient age. Telomerase expression determined by TRAP or immunohistochemistry was lower in stage I lung carcinomas (including 24 ADC, six SCC and four BC) than in other stages (II–IV) (*P*=0.03 for TRAP values and *P*=0.04 for hTERT scores) (Mann–Whitney *U* tests). However, no statistical evidence of an influence of levels of hTERT expression on survival was observed when all histological types were considered together or when SCLC and BC or SCC and ADC were considered separately. No correlation was observed between staining pattern and scores of protein expression, whatever the histological type and the disease stage considered. In contrast, a nucleolar pattern of hTERT staining, which was observed in four BC and 11 ADC out of 34 stage I NSCLC, correlated with a shorter survival (*P*=0.03) (Figure 3

).

## DISCUSSION

We have demonstrated here that telomerase expression varies significantly according to the histological type of lung tumours. Adenocarcinoma displayed the lowest level of telomerase activity especially at stage I and in the lepidic growth of mixed type ADC. This is in agreement with previous reports where telomerase activity in ADC ranged from 40 to 88% of positive cases, the lowest levels of expression being observed in bronchioloalveolar carcinoma (Hiyama K *et al*, 1995; Marchetti *et al*, 1999; Fujiwara *et al*, 2000) and in well-differentiated ADC of Clara cell type (Fujiwara *et al*, 2000). Indeed, we found a clear difference in the telomerase expression between the invasive compartment and the peripheral noninvasive lepidic growth of the mixed ADC with BAC predominant pattern.

In contrast, BC, which are truly aggressive lung tumours, exhibit similar high levels of telomerase expression than the SCLC, another well-known fast-growing tumour with 6 month mean survival. Basaloid carcinoma are rare tumours with a mean survival at stage I and II of 606 days as compared with 1218 days for SCC with which they had been confused (Brambilla, 1997). Telomerase activity in BC has never been reported yet since they represent a recent entity firstly described in 1992 (Brambilla *et al*, 1992) and now recognised in the new WHO histological classification of lung tumours in 1999 (Travis *et al*, 1999).

We observed a lower expression of hTERT in stage I NSCLC but no influence of telomerase levels of expression on survival rate. This point remains controversial (Hara *et al*, 2001; Toomey *et al*, 2001) although several authors strongly support the unfavourable prognostic value of a high telomerase expression in stage I NSCLC (Marchetti *et al*, 1999, 2002; Wang *et al*, 2002). Controversies regarding telomerase levels in tumours reside in the variety of technical approaches. Most previous data are based on telomerase activity measured by TRAP assay or quantification of hTERT mRNA by RT–PCR requiring samples containing at least 5000 viable tumour cells and obtained freshly in RNAse-free conditions. Sample contamination by telomerase negative normal epithelial or stromal cells might explain TRAP negative assay, whereas positive activated lymphocytes might contribute to a positive TRAP assay in the absence of telomerase activity in tumour cells (Cunningham *et al*, 1998; Xinarianos *et al*, 2000). A requirement of 70–80% of cancer cells seems reasonable in order to compare telomerase levels to external standards provided by cell lines (Marchetti *et al*, 2002). Furthermore, the exactitude of any measurement of telomerase activity is challenged by intratumoral heterogeneity of hTERT expression (Kumaki *et al*, 2001; Paradis *et al*, 2001). We and others have experienced successful hTERT *in situ* hybridisation approaches, which evaluate the level of transcription of hTERT (Kolquist *et al*, 1998; Soria *et al*, 2001; Wang *et al*, 2002). However, this technique remains time and labour-consuming. Therefore, the most promising tool for an *in situ* evaluation of telomerase expression is now represented by immunohistochemical detection of hTERT. To date, studies using noncommercially available antibodies have shown the nuclear expression of hTERT in tumour cells of various type, as well as in progenitor cells and activated lymphocytes and no expression in normal somatic cells. Among different commercially available antibodies against hTERT, only the monoclonal 44F12 antibody gave us both a unique and specific band on Western blotting and a clear-cut nuclear staining only in tumour component and activated lymphocytes. Furthermore, we found a high concordance between semiquantitative approaches of hTERT expression evaluated by immunohistochemistry and Western blotting on a same sample set.

A good correlation has been demonstrated between TRAP and hTERT immunohistochemical detection (Tahara *et al*, 1999; Kawakami *et al*, 2000; Kumaki *et al*, 2001, 2002) in colorectal tumours, liver tissues, lung cancer and mesothelioma. Our immunohistochemical approach in the setting of lung cancer was confronted to the TRAP assay as well as to hTERT *in situ* hybridisation and standard Western blotting. Similar profiles of RTA levels and hTERT staining scores were observed in lung tumours, higher levels being noted in SCLC and BC than in SCC and ADC. However in five cases where protein was detected by immunohistochemistry, telomerase activity was absent, and in 10 other cases, levels of telomerase expression evaluated by TRAP assay and immunohistochemistry were discordant. Such discrepancies might be explained by dilution of tumoral positive cells in the sample or by post-transcriptional and post-translational regulations of the protein quantitatively and qualitatively. As an example, the level of phosphorylation is able to control both telomerase activity (Li *et al*, 1997; Kang *et al*, 1999) and cytoplasmic *vs* nuclear localisation of hTERT (Kharbanda *et al*, 2000; Liu *et al*, 2001; Kyo and Inoue, 2002).

Interestingly, we reported for the first time a nucleolar localization of the catalytic subunit hTERT, preferentially located onto nucleolar structures in 45% of SCC and 42% of ADCs in contrast with its diffuse nuclear localization in all SCLC and 74% of BC. We have considered this pattern of staining as specific as it was observed in a number of TRAP and Western blot positive tumours. Indeed, compelling evidence has been provided that the assembly of hTERT subunit and hTERC RNA via box H/ACA motif takes place into the nucleolus favouring the hTERC maturation and the stabilization of the telomerase protein complex (Mitchell *et al*, 1999). Nucleolar localisation of hTERT seems also to occur independently of hTERC binding, suggesting that this phenomenon could correspond to a sequestration of hTERT away from its telomeric targets (Etheridge *et al*, 2002). In addition, subnuclear distribution of hTERT may vary according to cell cycle stage or DNA damage. Thus in normal cells, telomerase is released to the nucleoplasm during the S phase where it can add telomeric sequences to replicating chromosomes. In contrast, in response to ionising radiation, telomerase is excluded from the nucleoplasm and accumulated into the nucleolus in order to limit its accessibility to nontelomeric ends and to prevent its association to inappropriate substrates during the repair of NA breaks (Wong *et al*, 2002). Conversely in SV 40 transfected cells, oncogenic transformation triggers the releasing of hTERT into the nucleoplasmic compartment increasing the telomeric sequence synthesis (Wong *et al*, 2002). Since we report here a shorter survival in stage I NSCLC exhibiting a nucleolar pattern of staining, several hypotheses concerning the signification and the prognostic implication of nucleolar hTERT confinement may be proposed. The nucleolar localisation in some ADC and SCC is consistent with a regulated compartmentalised type of hTERT accumulation process where telomere elongation remains separated from DNA repair process during the S phase, thus protecting DNA from genetic instability and inopportune crisis (Wang *et al*, 2002). In contrast, concomitant nucleolar and nuclear distribution observed in aggressive tumours such as SCLC and BC is suggestive of a strong and aberrant increase of telomerase activation in fast-growing tumours that have acquired a large enough number of genetic lesions to escape senescence and apoptosis.

As telomerase inhibitors may be mainly effective after multiple cell divisions leading to cell death, they may have their greatest impact in combination with cytotoxic chemotherapy in advanced-stage disease and in high-grade tumours, such as BC and SCLC as well as in adjuvant therapy to surgery in early-stage disease. Although nucleolar localization in early NSCLC deserves specific attention, further studies need to be performed to improve our knowledge about telomerase regulation through its subnuclear distribution and cell cycle dependency in order to clarify the fundamental basis of the prognostic influence of hTERT nucleolar localisation.
